# False Positive Perfusion/Ventilation SPECT Study for Pulmonary Embolism in a Patient with Fontan Circulation

**DOI:** 10.4274/mirt.70883

**Published:** 2017-10-02

**Authors:** Emmanouil Panagiotidis

**Affiliations:** 1 Royal Liverpool University Hospital, Clinic of Nuclear Medicine, Liverpool, UK

**Keywords:** Fontan circulation, perfusion/ventilation, Single-photon emission computed tomography, Pulmonary embolism, computed tomography pulmonary angiogram

## Abstract

Fontan circulation is the consequence of an operation that results in the flow of systemic venous blood to the lungs without passing through a ventricle. An 18-year old man with a history of congenital heart disease surgically treated with Fontan circulation, presented with pleuritic chest pain and a raised D-dimer level. Perfusion/ventilation SPECT was performed to exclude the possibility of pulmonary embolism (PE) that showed unilateral reduced perfusion of the left lung with a mismatched right upper lobe defect, suspicious of PE. However, subsequent computed tomography pulmonary angiogram and clinical follow-up excluded the possibility of PE, emphasizing the need for knowledge of potential pitfalls to avoid false interpretations. Given the fact that adult congenital heart disease population is growing, with the majority having single ventricle/Fontan circulation and being at risk for thromboembolic disease, knowledge of the perfusion pattern pitfalls is important to avoid false interpretation and preventing the misdiagnosis of PE in patients with Fontan physiology.

## Abstract (tr)

Fontan dolaşım sistemik venöz kanın ventrikülden geçmeden akciğere ulaşmasına neden olan cerrahi girişime bağlıdır. Konjenital kalp hastalığı nedeniyle Fontan dolaşım cerrahisi geçirmiş 18 yaşında bir erkek hasta plöretik göğüs ağrısı ve yüksek D-dimer değeri ile başvurdu. Pulmoner emboliyi (PE) ekarte etmek amacıyla perfüzyon/ventilasyon SPECT yapıldı. PE için şüpheli olacak şekilde sol akciğerde unilateral perfüzyon azalması ve sağ üst lobda uyuşma defekti saptandı. Ancak bilgisayarlı tomografi pulmoner anjiyografi ve klinik takip ile PE olasılığı ekarte edildi. Çoğunluğu tek ventrikül/Fontan dolaşım sahibi ve dolayısıyla tromboembolik hastalık riski altında olan erişkin konjenital kalp hastalıklı popülasyonundaki artış göz önünde tutularak, Fontan fizyolojisi olan hastalarda PE yanlış tanısını ve yanlış yorumu engellemek için perfüzyon patern tuzaklarının bilinmesi önemlidir.

## Figures and Tables

**Figure 1:**
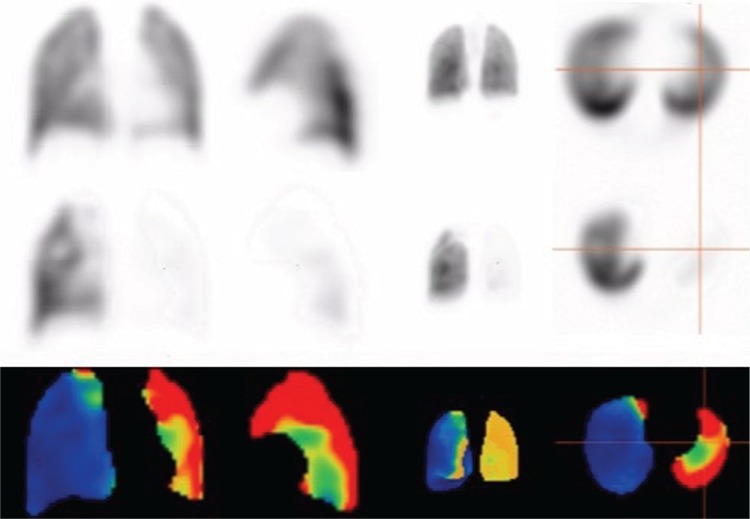
SPECT: First row: Ventilation with technegas, second row: Perfusion with 200 MBq of Tc-99m MAA, and third row: Subtraction of anterior, left lateral, MIP and transaxial images. There is satisfactory ventilation in both lungs. However, there is nearly no perfusion in the left lung (cross in transaxial images). Moreover, there is a subsegmental perfusion/ventilation mismatched defect in the right upper lobe, raising the possibility of pulmonary embolism (PE)

**Figure 2:**
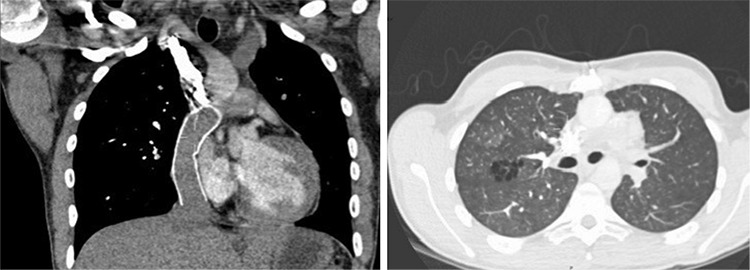
Computed tomography (CT) pulmonary angiogram coronal and transaxial images below the level of the carina. A extra cardiac conduit demonstrated with the superior vena cava (SVC) to pulmonary arteries (PA) and a stent within the intra-thoracic inferior vena cava (IVC) which enters the right PA resulting to single ventricle/Fontan physiology. There is non-opacified blood within the stent as a result of passive venous supply to the lungs in Fontan circulation and IV contrast seen within the stent post upper extremity contrast administration and passive venous supply to the lungs. There is also a hypoplastic right ventricle. The peripheral PA are patent with no evidence of distal PE. There is ground glass shadowing in the right upper lobe with minor cystic changes, which could represent an atypical infection. The Fontan procedure refers to any surgical procedure that leads to systemic flow of venous blood to the lungs without passing through a ventricle. In 1971, Fontan and Baudet (1) described a surgical procedure for repair of tricuspid atresia that was built on experimental and clinical research since the 1940s. The principle of the Fontan operation is diversion of systemic venous return directly to the PA, thus by-passing the right ventricle when the latter is nonexistent, too small, or dysfunctional (2). Thromboembolism can be a significant cause of morbidity and mortality after the Fontan operation, with contributing risk factors including low flow state, stasis in venous pathways, right to left shunt, blind cul de sacs, prosthetic materials and/or arrhythmias (3,4,5). It has been demonstrated that the prevalence of silent PE in adult patients with Fontan circulation was 17%, while the long-term hemodynamic implications of this with respect to Fontan attrition over time have been unknown (3). Fontan circulation can mimic PE on perfusion lung imaging. Unilaterally decreased relative lung perfusion is the most common perfusion abnormality seen in patients with congenital heart disease such as congenital absence of PA, wherein ipsilateral lung perfusion occurs through collaterals from bronchial arteries that cannot be assessed by lung perfusion except in patients with a functioning right to left shunt (6). Other differential diagnoses of unilateral absence of lung perfusion include pulmonary aplasia (absence of ipsilateral PA, absence of ipsilateral pulmonary tissue, and bronchus terminating in dilated blind pouch), hyperlucent lung syndrome, and tetralogy of Fallot (7). However, the most common pattern in perfusion scan of congenital heart disease is unilateral decreased lung perfusion, as in our case (8). Several methods to minimize misdiagnosis of PE in Fontan patients have been proposed. Misinformation regarding pulmonary flow patterns can occur because of preferential blood flow from the SVC to the right PA and from the IVC to the left PA. A proposed protocol to resolve this problem includes injection of macroaggregates for perfusion scanning into the arm and any foot vein. Moreover, the timing of image acquisition with contrast during CT pulmonary angiography is also critical to prevent false positive diagnoses. Given the fact that adult congenital heart disease population is growing, with the majority having single ventricle/Fontan circulation and being at risk for thromboembolic disease, knowledge of the perfusion pattern pitfalls is important to avoid false interpretation and preventing the misdiagnosis of PE in patients with Fontan physiology
